# Targeting L-Proline Uptake as New Strategy for Anti-chagas Drug Development

**DOI:** 10.3389/fchem.2020.00696

**Published:** 2020-08-25

**Authors:** Lucía Fargnoli, Esteban A. Panozzo-Zénere, Lucas Pagura, María Julia Barisón, Julia A. Cricco, Ariel M. Silber, Guillermo R. Labadie

**Affiliations:** ^1^Facultad de Ciencias Bioquímicas y Farmacéuticas, Instituto de Química de Rosario (IQUIR), Universidad Nacional de Rosario, Rosario, Argentina; ^2^Instituto de Biología Molecular y Celular de Rosario (IBR), Consejo Nacional de Investigaciones Científicas y Técnicas CONICET–Facultad de Ciencias Bioquímicas y Farmacéuticas, Universidad Nacional de Rosario, Rosario, Argentina; ^3^Laboratory of Biochemistry of Tryps-LaBTryps, Departamento de Parasitologia, Instituto de Ciências Biomédicas, Universidade de São Paulo, Cidade Universitária, São Paulo, Brazil; ^4^Departamento de Química Orgánica, Facultad de Ciencias Bioquímicas y Farmacéuticas, Universidad Nacional de Rosario, Rosario, Argentina

**Keywords:** Chagas disease, proline uptake, *T. cruzi* epimastigotes, cytotoxicity, target validation

## Abstract

L-Proline is an important amino acid for the pathogenic protists belonging to *Trypanosoma* and *Leishmania* genera. In *Trypanosoma cruzi*, the etiological agent of Chagas disease, this amino acid is involved in fundamental biological processes such as ATP production, differentiation of the insect and intracellular stages, the host cell infection and the resistance to a variety of stresses. In this study, we explore the L-Proline uptake as a chemotherapeutic target for *T. cruzi*. Novel inhibitors have been proposed containing the amino acid with a linker and a variable region able to block the transporter. A series of sixteen 1,2,3-triazolyl-proline derivatives have been prepared for *in vitro* screening against *T. cruzi* epimastigotes and proline uptake assays. We successfully obtained inhibitors that interfere with the amino acid internalization, which validated our design targeting the metabolite's transport. The presented structures are one of few examples of amino acid transporter inhibitors. The unprecedent application of this strategy on the development of new chemotherapy against Chagas disease, opens a new horizon on antiparasitic drug development against parasitic diseases and other pathologies.

## Introduction

Chagas disease is one of the most neglected infectious disease. It is endemic in the Americas, with 8–10 million people infected and 25 million people at risk (Nunes et al., 2013). The disease is divided in the acute and the chronic phase. The first have a noticeable parasitemia, the absence of humoral response and is largely asymptomatic. The chronic phase has a non-evident parasitemia and a robust IgG response being asymptomatic in 60–70% of the cases (Pérez-Molina and Molina, 2018). The chemotherapy against Chagas disease relies mainly on two drugs introduced more than 40 years ago: Nifurtimox (Nf) and Benznidazole (Bz) (Urbina, 2010). Both drugs are efficient on the acute phase, but in the chronic phase is controversial (Morillo et al., 2015). In addition, severe side effects due to toxicity and the emergence of resistance calls for urgent development of new drugs (Guedes et al., 2011).

*Trypanosoma cruzi* is a hemoflagellated parasite that causes Chagas disease. This parasite presents a complex life cycle among two kinds of hosts: mammals and reduviid insects, which transmit the infection. Along its life-cycle at least four stages were clearly identified, epimastigotes (replicative stage) and metacyclic trypomastigotes (infective, non-replicative stage) in the insect and blood stream trypomastigotes and amastigotes (intracellular replicative stage). Also, during its life-cycle the parasite faces different environments and it must adjust its “life-style” including its metabolism to changes in nutrients availability, temperature, together other environmental variables.

Among many other important metabolites, amino acids are particularly relevant for the biology of *T. cruzi*, besides protein synthesis (Marchese et al., 2018), playing fundamental roles in energy management (Pereira et al., 2000) and nitrogen metabolism (Crispim et al., 2018; Girard et al., 2018). When epimastigotes proliferation arrests, there is a metabolic switch from a carbohydrates to an amino acids based metabolism, with a consequent change in the protein expression profile (Barisón et al., 2017; Avila et al., 2018). In fact, it has been demonstrated that amino acids such as proline (Paes et al., 2013), histidine (Barison et al., 2016), and even alanine (Girard et al., 2018), as well as the proline oxidation product P5C (Mantilla et al., 2015), can fuel electrons to the respiratory chain, powering the mitochondrial ATP synthesis (Sylvester and Krassner, 1976; Martins et al., 2009; Paes et al., 2013; Barison et al., 2016). Some neutral amino acids can also function as osmolytes, serving to counteract volume perturbations following a shift in extracellular osmolarity (Rohloff et al., 2003; Silber et al., 2005; Avila et al., 2018).

Particularly, proline is involved in energization of the host-cells invasion by metacyclic trypomastigotes (Martins et al., 2009), as well as growth and differentiation of the insect (Contreras Vt et al., 1988; Tonelli et al., 2004; Silber et al., 2009) and the intracellular stages (Tonelli et al., 2004). Additionally, its accumulation in the parasite cytoplasm provides resistance to oxidative and thermal stress (Tonelli et al., 2004; Magdaleno et al., 2009; Paes et al., 2013; Sayé et al., 2014). The proline availability is mediated by an interplay of the biosynthesis degradation and uptake process (Sylvester and Krassner, 1976; Silber et al., 2002; Magdaleno et al., 2009; Paes et al., 2013; Sayé et al., 2014; Mantilla et al., 2017). In particular, the inhibition of proline uptake by competitive transporter interrupters, diminished the parasites ability to tolerate oxidative imbalance, nutritional stress and to complete the infection cycle (Magdaleno et al., 2009).

Taking the proline uptake as a novel drug target we decided to develop new transporter inhibitors and evaluate their antiproliferative activity against *Trypanosoma cruzi*. These new compounds were initially evaluated on *T. cruzi* epimastigotes, validating their action mechanism by proline transport experiments. A comprehensive analysis of the structure-activity relationship allowed a rational pipeline to design selective metabolite transporter inhibitors.

## Materials and Methods

### Chemistry

#### General Remarks

Chemical reagents were purchased from commercial suppliers and used without further purification, unless otherwise noted. Dry, deoxygenated diethyl ether (Et_2_O), tetrahydrofuran (THF), and dichloromethane (DCM) were obtained bypassing commercially available pre-dried, oxygen-free solvents through activated alumina columns. DMF was distilled from BaO. Reactions were monitored by thin-layer chromatography (TLC) performed on 0.2 mm Merck silica gel aluminum plates (60F-254) and visualized using ultraviolet light (254 nm) and by potassium permanganate and heat as developing reagents. All reactions were performed under an atmosphere of nitrogen using oven-dried glassware and standard syringe/septa techniques. Column chromatography was performed with silica gel 60 (230–400 mesh). Yields were calculated for material judged homogeneous by thin layer chromatography (TLC) and nuclear magnetic resonance (^1^H NMR).

^1^H and ^13^C NMR spectra were acquired on a Bruker Avance II 300 MHz (75.13 MHz) using CDCl_3_ as solvent. Chemical shifts (δ) were reported in ppm downfield from tetramethylsilane and coupling constants are in hertz (Hz). NMR spectra were obtained at 298 K unless otherwise stated and samples run as a dilute solution of the stated solvent. All NMR spectra were referenced to the residual undeuterated solvent as an internal reference. The following abbreviations were used to explain the multiplicities: s = singlet, d = doublet, t = triplet, q = quartet, m = multiplet, br = broad. Assignment of proton resonances was confirmed by correlated spectroscopy. Electrospray ionization high-resolution mass spectra (ESI-HRMS) were recorded on a Bruker MicroTOF II. IR spectra were obtained using an FT-IR Shimadzu spectrometer and only partial spectral data are listed. Melting points were measured on an Electrothermal 9100 apparatus and are uncorrected.

### Experimental Procedures and Spectroscopic Data

#### Synthesis of N-Propargyl Methyl Prolinate (2)

To a solution of methyl prolinate (200 mg, 1.22 mmol) in 10 mL of Et_2_O_(anh)_, NEt_3_ (439 mg, 4.3 mmol) and 80 % propargyl bromide in toluene (263 μL, 2.45 mmol) were added in this order and the reaction mixture was stirred at room temperature for 12 h. The solvent was evaporated, and the crude product was purified by column chromatography in silica gel with increasing ethyl acetate/hexane gradient to yield the expected product as a yellow oil (129 mg, 72 %).

^1^H NMR (300 MHz, CDCl_3_): δ 3.72 (s, 3H, OCH_3_), 3.61–3.55 (m, 2H, C5-H, C2-H), 3.45–3.39 (m, 1H, C6-H), 3.07–3.01 (m, 1H, C6-H), 2.75–2.67 (m, 1H, C5-H), 2.20−2.06 (m, 2H, C7-H and C3-H), 2.08–1.76 (m, 3H, C3-H, C4-H). ^13^C NMR (75 MHz, CDCl_3_): δ 173.9 (COO), 78.2 (C), 73.2 (CH), 62.4 (CH), 52.1 (CH_2_), 51.9 (OCH_3_), 41.1 (CH_2_), 29.5 (CH_2_), 23.2 (CH_2_).

#### General Procedure for the Cu(I) Mediated 1,3-Dipolar Cycloaddition

N-Propargyl methyl prolinate (1 eq) and the azide (1.1 eq) were suspended in 10 mL/eq of *t*BuOH:H_2_O (1:1) and then 1M CuSO_4_ solution (0.05 eq) and finally 1M sodium ascorbate solution (0.2 eq) and the mixture stirred overnight at room temperature. Brine was added, and the solution was extracted with dichloromethane. Combined organic extracts were dried over sodium sulfate and evaporated. Products were purified by column chromatography in silica gel with increasing hexanes/ethyl acetate/methanol gradients.

#### Methyl ((1-(2-Ethoxy-2-Oxoethyl)-1H-1,2,3-Triazol-4-yl)Methyl)-L-Prolinate (3a)

Compound **3a** was prepared from 45 mg (0.27 mmol) of N-propargyl methyl prolinate **1**, following the general procedure for the 1,3-dipolar cycloaddition, affording 66 mg of a yellowish oil in 80 % yield. ^1^H NMR (300 MHz, CDCl_3_): δ 7.62 (s, 1H, C7-H), 5.09 (d, *J* = 2.6 Hz, 2H, C8-H), 4.20 (q, *J* = 7.5 Hz, 2H, CH_2_CH_3_), 4.01 (d, *J* = 13.8 Hz, 1H, C6-H), 3.82 (d, *J* = 13.8 Hz, 1H, C6-H), 3.64 (s, 3H, OMe), 3.29 (dd, *J* = 8.7, 6.1 Hz, 1H, C2-H), 3.10–3.04 (m, 1H, C5-H), 2.50 (dt, *J* = 8.4 Hz, *J* = 8.2 Hz, 1H, C5-H), 2.07–1.74 (m, 4H, C3-H and C4-H), 1.23 (t, *J* = 7.5 Hz, 3H, CH_2_CH_3_). ^13^C NMR (75 MHz, CDCl_3_): δ 174.1 (COO), 166.3 (COO), 144.5 (C6'), 124.4 (C7), 64.4 (C2), 62.4 (CH_2_), 53.0 (C5), 52.0 (OCH_3_), 50.8 (C8), 48.2 (C6), 29.3 (C3), 23.0 (C4), 14.0 (CH_3_). IR (film): υ_max_ 3458, 3439, 2954, 2357, 1732, 1643, 1444, 1217, 1051, 1024, 875, 798, 756 cm^−1^. ESI-HRMS *m/z* [M+K]^+^ calcd for C_13_H_20_KN_4_O_4_ 335.1116, found 335.1113.

#### Methyl ((1-(5-Ethoxy-5-Oxopentyl)-1H-1,2,3-Triazol-4-yl)Methyl)-L-Prolinate (3b)

Compound **3b** was prepared from 45 mg (0.27 mmol) of N-propargyl methyl prolinate **1**, following the general procedure for the 1,3-dipolar cycloaddition, affording 63 mg of a yellowish oil in 69 % yield. ^1^H NMR (300 MHz, CDCl_3_): δ 7.50 (s, 1H, C7-H), 4.38 (d, *J* = 2.6 Hz, 2H, C8-H), 4.09 (q, *J* = 7.5 Hz, 2H, **CH**_**2**_CH_3_), 3.97 (d, *J* = 13.8 Hz, 1H, C6-H), 3.79 (d, *J* = 13.8 Hz, 1H, C6-H), 3.65 (3H, OMe), 3.28 (dd, *J* = 8.7, 6.1 Hz, 1H, C2-H), 3.12–3.07 (m, 1H, C5-H), 2.53–2.45 (m, 1H, C5-H), 2.32–2.08 (m, 6H, CH_2_), 1.91–1.76 (m, 4H, C3-H and C4-H), 1.20 (t, *J* = 7.5 Hz, 3H, CH_2_**CH**_**3**_). ^13^C NMR (75 MHz, CDCl_3_): δ 174.4 (C=O), 172.3 (COO), 144.5 (C6'), 122.8 (C7), 64.6 (C2), 60.6 (**CH**_**2**_CH_3_), 53.3 (C5), 51.8 (OCH_3_), 49.1 (C8), 48.5 (C6), 30.6 (CH_2_), 29.6 (CH_2_), 29.3 (C3), 25.4 (C4), 23.0 (CH_2_), 14.1 (CH_2_**CH**_**3**_). IR (film): υ_max_ 3437, 3138, 2954, 2358, 1730, 1633, 1444, 1377, 1348, 1274, 1199, 1047, 1028, 854, 802 cm^−1^. ESI-HRMS *m/z* [M+H]^+^ calcd for C_16_H_27_N_4_O_4_ 339.2027, found 339.2029.

#### Methyl ((1-Benzyl-1H-1,2,3-Triazol-4-yl)Methyl)-L-Prolinate (3c)

Compound **3c** was prepared from 45 mg (0.27 mmol) of N-propargyl methyl prolinate **1**, following the general procedure for the 1,3-dipolar cycloaddition, affording 66 mg of a yellowish oil in 80 % yield. ^1^H NMR (300 MHz, CDCl_3_): δ 7.42 (s, 1H, C7-H), 7.36–7.30 (m, 3H, Ph), 7.27–7.21 (m, 2H, Ph), 5.48 (d, *J* = 2.6 Hz, 2H, C8-H), 3.95 (d, *J* = 13.8 Hz, 1H, C6-H), 3.78 (d, *J* = 13.8 Hz, 1H, C6-H), 3.60 (s, 3H, OMe), 3.27 (dd, *J* = 8.7, 6.1 Hz, 1H, C2-H), 3.09 (m, 1H, C5-H), 2.48 (dt, *J* = 8.4, 8.2 Hz, 1H, C5-H), 2.18–1.66 (m, 4H, C3-H and C4-H). ^13^C NMR (75 MHz, CDCl_3_): δ 174.6 (COO), 145.1 (C6'), 134.8 (Ph, C), 129.2 (Ph, CH), 128.8 (Ph, CH), 128.3 (Ph, CH), 122.8 (C7), 64.9 (C2), 54.2 (C5), 53.5 (C8), 51.9 (OCH_3_), 48.9 (C6), 29.5 (C3), 23.2 (C4). IR (film): υ_max_ 3493, 3140, 2951, 2850, 2359, 1745, 1732, 1556, 1496, 1454, 1359, 1284, 1049, 769, 725 cm^−1^. ESI-HRMS *m/z* [M+Na]^+^ calcd for C_16_H_20_N_4_NaO_2_ 323.1478, found 323.1474.

#### Methyl ((1-Cyclohexyl-1H-1,2,3-Triazol-4-yl)Methyl)-L-Prolinate (3d)

Compound **3d** was prepared from 45 mg (0.27 mmol) of N-propargyl methyl prolinate **1**, following the general procedure for the 1,3-dipolar cycloaddition, affording 31 mg of a yellowish oil in 39% yield. ^1^H NMR (300 MHz, CDCl_3_): δ 7.51 (s, 1H, C7-H), 4.44 (d, *J* = 2.6 Hz, 2H, C8-H), 3.97 (d, *J* = 13.8 Hz, 1H, C6-H), 3.79 (d, *J* = 13.8 Hz, 1H, C6-H), 3.66 (s, 3H, OMe), 3.29 (dd, *J* = 8.7, 6.1 Hz, 1H, C2-H), 3.16–3.10 (m, 1H, C5-H), 2.46 (dt, *J* = 8.4, 8.2 Hz, 1H, C5-H), 2.20–1.63 (m, 12H, C3-H and cyclohexyl), 1.51–1.21 (m, 3H, C4-H and cyclohexyl). ^13^C NMR (75 MHz, CDCl_3_): δ 174.5 (COO), 144.0 (C6'), 120.3 (C7), 64.8 (C2), 59.7 (C5), 53.4 (C8), 51.85 (OCH_3_), 48.9 (C6), 33.5 (CH_2_), 32.4 (C3), 29.4 (CH_2_), 25.2 (CH_2_), 23.0 (C4). IR (film): υ_max_ 3417, 3142, 2935, 2856, 2362, 1737, 1732, 1633, 1450, 1371, 1276, 1201, 1049, 997, 894, 823, 777 cm^−1^. ESI-HRMS *m/z* [M+H]^+^ calcd for C_15_H_25_N_4_O_2_ 293.1972, found 293.1970.

#### Methyl ((1-(3-Phenylpropyl)-1H-1,2,3-Triazol-4-yl)Methyl)-L-Prolinate (3e)

Compound **3e** was prepared from 45 mg (0.27 mmol) of N-propargyl methyl prolinate **1**, following the general procedure for the 1,3-dipolar cycloaddition, affording 73 mg of a yellowish oil in 82% yield. ^1^H NMR (300 MHz, CDCl_3_): δ 7.43 (s, 1H, C7-H), 7.24–7.19 (m, 2H, Ph), 7.18–7.08 (m, 3H, Ph), 4.21 (d, *J* = 2.6 Hz, 2H, C8-H), 3.93 (d, *J* = 13.8 Hz, 1H, C6-H), 3.76 (d, *J* = 13.8 Hz, 1H, C6-H), 3.60 (s, 3H, OMe), 3.24 (dd, *J* = 8.7, 6.1 Hz, 1H, C2-H), 3.06 (m, 1H, C5-H), 2.53–2.44 (m, 5H), 2.23–1.61 (m, 4H, C3-H and C4-H). ^13^C NMR (75 MHz, CDCl_3_): δ 174.5 (COO), 144.6 (C6'), 140.3 (Ph), 128.7 (Ph), 128.5 (Ph), 126.4 (Ph), 122.8 (C7), 64.7 (C2), 53.4 (C5), 51.9 (OCH_3_), 49.5 (C8), 48.7 (C6), 32.5 (CH_2_), 31.7 (C3), 29.5 (CH_2_), 23.5 (C4). IR (film): υ_max_ 3626, 3458, 3138, 2949, 2854, 2358, 1732, 1602, 1496, 1444, 1354, 1278, 1172, 1085, 1049, 1004, 785, 748, 702 cm^−1^. ESI-HRMS *m/z* [M+Na]^+^ calcd for C_18_H_24_N_4_NaO_2_ 351.1791, found 351.1790.

#### Methyl ((1-Cinnamyl-1H-1,2,3-Triazol-4-yl)Methyl)-L-Prolinate (3f)

Compound **3f** was prepared from 45 mg (0.27 mmol) of N-propargyl methyl prolinate **1**, following the general procedure for the 1,3-dipolar cycloaddition, affording 53 mg of a yellowish oil in 60% yield. ^1^H NMR (300 MHz, CDCl_3_): δ 7.58 (s, 1H, C7-H), 7.43–7.21 (m, 5H, Ph), 6.65 (d, *J* = 15.8 Hz, 1H, C9-H), 6.33 (m, 1H, C10-H), 5.11 (d, *J* = 2.6 Hz, 2H, C8-H), 4.00 (d, *J* = 13.8 Hz, 1H, C6-H), 3.82 (d, *J* = 13.8 Hz, 1H, C6-H), 3.66 (s, 3H, OMe), 3.31 (dd, *J* = 8.7, 6.1 Hz, 1H, C2-H), 3.14 (m, 1H, C5-H), 2.52 (dt, *J* = 8.4, 8.2 Hz, 1H, C5-H), 2.20–2.04 (m, 1H, C3-H), 2.00–1.71 (m, 3H, C3-H, C4-H_2_). ^13^C NMR (75 MHz, CDCl3): δ 174.5 (COO), 144.9 (C6'), 135.3 (Ph), 135.3 (C9-H), 128.7 (Ph), 128.5 (CH), 126.7 (Ph), 122.5 (C10-H), 121.9 (C7), 64.7 (C2), 53.4 (C5), 52.3 (OCH_3_), 51.8 (C8), 48.7 (C6), 29.4 (C3), 23.0 (C4). IR (film): υ_max_ 3541, 3138, 2951, 2845, 2358, 1741, 1732, 1552, 1448, 1359, 1278, 1203, 1174, 1128, 1047, 970, 756, 694 cm^−1^. ESI-HRMS *m/z* [M+Na]^+^ calcd for C_18_H_22_N_4_NaO_2_ 349.1635, found 349.1625.

#### Methyl ((1-(Naphthalen-2-Ylmethyl)-1H-1,2,3-Triazol-4-yl)Methyl)-L-Prolinate (3g)

Compound **3g** was prepared from 20 mg (0.12 mmol) of N-propargyl methyl prolinate **1**, following the general procedure for the 1,3-dipolar cycloaddition, affording 34 mg of a light orange solid in 81% yield. M.p. 64.0-64.9 °C. ^1^H NMR (300 MHz, CDCl_3_): δ 7.85–7.82 (m, 3H, naphtyl), 7.74 (s, 1H, C-H), 7.52–7.49 (m, 2H, naphtyl), 7.46 (s, 1H, C7-H), 7.37–7.33 (m, 1H, naphtyl), 5.66 (d, *J* = 3.6 Hz, 2H, C8-H), 3.97 (d, *J* = 13.9 Hz, 1H, C6-H), 3.80 (d, *J* = 13.9 Hz, 1H, C6-H), 3.60 (s, 3H, OMe), 3.26 (dd, *J* = 8.7, 6.1 Hz, 1H, C2-H), 3.06 (m, 1H, C5-H), 2.46 (dt, *J* = 8.4, 8.2 Hz, 1H, C5-H), 2.15–1.65 (m, 4H, C3-H and C4-H). ^13^C NMR (75 MHz, CDCl3): δ 174.4 (COO), 145.1 (C6'), 133.2 (C), 133.1 (C), 131.9 (C), 129.1 (CH), 127.9 (CH), 127.7 (CH), 127.4 (CH), 126.7 (CH), 125.3 (C7), 122.7 (CH), 122.7 (CH), 64.7 (C2), 54.3 (C5), 53.4 (C8), 51.8 (OCH_3_), 43.7 (C6), 29.4 (C3), 23.0 (C4). IR (KBr): υ_max_ 3500, 3132, 2949, 2818, 2358, 1732, 1600, 1548, 1508, 1435, 1338, 1273, 1203, 1172, 1126, 1047, 891, 771 cm^−1^. ESI-HRMS *m/z* [M+H]^+^ calcd for C_20_H_23_N_4_O_2_ 351.1815, found 351.1826.

#### Methyl ((1-Octyl-1H-1,2,3-Triazol-4-yl)Methyl)-L-Prolinate (3h)

Compound **3h** was prepared from 20 mg (0.12 mmol) of N-propargyl methyl prolinate **1**, following the general procedure for the 1,3-dipolar cycloaddition, affording 31 mg of a yellow oil in 81% yield. ^1^H NMR (300 MHz, CDCl_3_): δ 7.44 (s, 1H, C7-H), 4.24 (t, *J* = 7.2 Hz, 2H, C8-H), 3.93 (d, *J* = 13.8 Hz, 1H, C6-H), 3.75 (d, *J* = 13.8 Hz, 1H, C6-H), 3.61 (s, 3H, OMe), 3.23 (dd, *J* = 8.6, 6.0 Hz, 1H, C2-H), 3.08–3.02 (m, 1H, C5-H), 2.49–2.41 (dt, 1H, *J* = 8.4, 8.1 Hz, C5-H), 2.03–1.71 (m, 5H), 1.22–1.17 (m, 11H), 0.79 (t, *J*= 6.5 Hz, 3H, CH_3_). ^13^C NMR (75 MHz, CDCl_3_): δ 174.4 (COO), 144.4 (C6'), 122.5 (C7), 64.6 (C2), 53.2 (C5), 51.8 (OCH_3_), 50.2 (C8), 48.6 (C6), 31.6 (CH_2_), 30.2 CH_2_), 29.4 (C3), 29.0 (CH_2_), 28.9 (CH_2_), 26.4 (CH_2_), 23.0 (C4), 22.5 (CH_2_), 14.0 (CH_3_). IR (film): υ_max_ 3604, 3458, 3136, 2926, 2357, 1345, 1645, 1444, 1354, 1172, 1047, 771 cm^−1^. ESI-HRMS *m/z* [M+Na]^+^ calcd for C_17_H_30_N_4_NaO_2_ 345.2261, found 345.2261.

#### Methyl ((1-Decyl-1H-1,2,3-Triazol-4-yl)Methyl)-L-Prolinate (3i)

Compound **3i** was prepared from 20 mg (0.12 mmol) of N-propargyl methyl prolinate **1**, following the general procedure for the 1,3-dipolar cycloaddition, affording 32 mg of a yellow oil in 75% yield. ^1^H NMR (300 MHz, CDCl_3_): δ 7.49 (s, 1H, C7-H), 4.32 (t, *J* = 7.2 Hz, 2H, C8-H), 4.02 (d, *J* = 13.8 Hz, 1H, C6-H), 3.83 (d, *J* = 13.8 Hz, 1H, C6-H), 3.66 (s, 3H, OMe), 3.33 (dd, *J* = 8.7, 6.1 Hz, 1H, C2-H), 3.17–3.08 (m, 1H, C5-H), 2.58–2.50 (dt, *J* = 8.4, 8.2 Hz, 1H, C5-H), 2.11–1.77 (m, 4H, C3-H and C4-H), 1.27–1.21 (m, 16H), 0.84 (t, *J* = 6.6 Hz, 3H, CH_3_). ^13^C NMR (75 MHz, CDCl_3_): δ 174.5 (COO), 144.4 (C6'), 122.7 (C7), 64.7 (C2), 53.4 (C5), 51.9 (OCH_3_), 50.4 (C8), 48.7 (C6), 31.9 (CH_2_), 30.4 (CH_2_), 29.5 (C3), 29.5 (CH_2_), 29.3 (CH_2_), 29.1 (CH_2_), 29.1 (CH_2_), 26.5 (CH_2_), 23.1 (C4), 22.7 (CH_2_), 14.2 (CH_3_). IR (film): υ_max_ 3458, 2926, 2854, 2358, 1745, 1732, 1651, 1444, 1373, 1278, 1199, 1172, 1047, 891, 783, 721 cm^−1^. ESI-HRMS *m/z* [M+H]^+^ calcd for C_19_H_35_N_4_O_2_ 351.2754, found 351.2746.

#### Methyl ((1-Hexadecyl-1H-1,2,3-Triazol-4-yl)Methyl)-L-Prolinate (3j)

Compound **3j** was prepared from 20 mg (0.12 mmol) of N-propargyl methyl prolinate **1**, following the general procedure for the 1,3-dipolar cycloaddition, affording 22 mg of a white solid in 42 % yield. M.p. 60-60.9 °C. ^1^H NMR (300 MHz, CDCl_3_): δ 7.47 (s, 1H, C7-H), 4.29 (t, *J* = 7.2 Hz, 2H, C8-H), 3.99 (d, *J* = 13.8 Hz, 1H, C6-H), 3.80 (d, *J* = 13.8 Hz, 1H, C6-H), 3.67 (s, 3H, OMe), 3.30 (dd, *J* = 8.7, 6.1 Hz, 1H, C2-H), 3.16–3.11 (m, 1H, C5-H), 2.57–2.48 (dt, *J* = 8.9, 7.8 Hz, 1H, C5- H), 1.91–1.84 (m, 4H), 1.28–1.23 (m, 28H), 0.85 (t, *J* = 6.6 Hz, 3H, CH_3_). ^13^C NMR (75 MHz, CDCl_3_): δ 174.7 (COO), 144.7 (C6'), 122.6 (C7), 64.6 (C2), 53.2 (C5), 52.0 (OCH_3_), 50.5 (C8), 48.9 (C6), 32.1 (CH_2_), 30.5 (CH_2_), 29.9 (CH_2_), 29.8 (CH_2_), 29.8 (CH_2_), 29.7 (CH_2_), 29.6 (CH_2_), 29.6 (CH_2_), 29.6 (CH_2_), 29.4 (C3), 29.3 (CH_2_), 29.2 (CH_2_), 28.9 (CH_2_), 26.4 (CH_2_), 23.0 (C4), 22.6 (CH_2_), 14.0 (CH_3_). IR (KBr): υ_max_ 3124, 2914, 2357, 1745, 1728, 1556, 1444, 1336, 1269, 1197, 1053, 848, 790, 719 cm^−1^. ESI-HRMS *m/z* [M+Na]^+^ calcd for C_25_H_46_N_4_NaO_2_ 457.3513, found 457.3512.

#### Methyl (Z)-((1-(Octadec-9-en-1-yl)-1H-1,2,3-Triazol-4-yl)Methyl)-L-Prolinate (3k)

Compound **3k** was prepared from 20 mg (0.12 mmol) of N-propargyl methyl prolinate **1**, following the general procedure for the 1,3-dipolar cycloaddition, affording 33 mg of a light-yellow oil in 59 % yield. ^1^H NMR (300 MHz, CDCl_3_): δ 7.49 (s, 1H, C7-H), 5.35–5.31 (m, CH=CH, 2H), 4.30 (t, *J* = 7.2 Hz, 2H, C8-H), 4.00 (d, *J* = 13.8 Hz, 1H, C6-H), 3.81 (d, *J* = 13.8 Hz, 1H, C6-H), 3.68 (s, 3H, OMe), 3.30 (dd, *J* = 8.8, 6.1 Hz, 1H, C2-H), 3.15–3.10 (m, 1H, C5-H), 2.56–2.48 (dt, *J* = 8.5, 8.1 Hz, 1H, C5-H), 2.16–1.79 (m, 6H), 1.27–1.25 (m, 26H), 0.87 (t, *J* = 6.7 Hz, 3H, CH_3_). ^13^C NMR (75 MHz, CDCl_3_): δ 174.4 (COO), 144.4 (C6'), 130.0 (CH=), 129.7 (CH=), 122.5 (C7), 64.7 (C2), 53.3 (C5), 51.8 (OCH_3_), 50.3 (C8), 48.6 (C6), 31.9 (CH_2_), 30.3 (CH_2_), 29.7 (CH_2_), 29.7 (CH_2_), 29.5 (C3), 29.4 (CH_2_), 29.4 (CH_2_), 29.3 (CH_2_), 29.1 (CH_2_), 29.0 (CH_2_), 29.0 (CH_2_), 27.2 (CH_2_), 27.1 (CH_2_), 26.5 (CH_2_), 23.0 (C4), 22.6 (CH_2_), 14.1 (CH_3_). IR (film): υ_max_ 3564, 3477, 3136, 2924, 2852, 2358, 2096, 1745, 1556, 1444, 1373, 1276, 1199, 1172, 1047, 968, 891, 775, 723 cm^−1^. ESI-HRMS *m/z* [M+K]^+^ calcd for C_27_H_48_ KN_4_O_2_ 499.3408, found 499.3412.

#### Methyl ((1-Icosyl-1H-1,2,3-Triazol-4-yl)Methyl)-L-Prolinate (3l)

Compound **3l** was prepared from 20 mg (0.12 mmol) of N-propargyl methyl prolinate **1**, following the general procedure for the 1,3-dipolar cycloaddition, affording 34 mg of a white solid in 58 % yield. M.p. 72.7–73.7 °C. ^1^H NMR (300 MHz, CDCl_3_): δ 7.49 (s, 1H, C7-H), 4.31 (t, *J* = 7.2 Hz, 2H, C8-H), 4.01 (d, *J* = 13.8 Hz, 1H, C6-H), 3.82 (d, *J* = 13.8 Hz, 1H, C6-H), 3.69 (s, 3H, OMe), 3.30 (dd, *J* = 8.5, 5.7 Hz, 1H, C2-H), 3.14–3.1 (m, 1H, C5-H), 2.54–2.46 (dt, *J* = 8.5, 7.9 Hz, 1H, C5- H), 1.91–1.84 (m, 4H), 1.28–1.23 (m, 36H), 0.87 (t, *J* = 6.7 Hz, 3H, CH_3_). ^13^C NMR (75 MHz, CDCl_3_): δ 174.5 (COO), 144.4 (C6'), 122.4 (C7), 64.7 (C2), 53.3 (C5), 51.8 (OMe), 50.2 (C8), 48.6 (CH_2_), 31.9 (CH_2_), 30.2 (CH_2_), 29.6 (CH_2_), 29.5 (CH_2_), 29.3 (CH_2_), 29.0 (CH_2_), 26.4 (CH_2_), 23.0 (CH_2_), 22.6 (CH_2_), 14.1 (CH_3_). IR (KBr): υ_max_ 3649, 3124, 3076, 2916, 2846, 2358, 1743, 1462, 1338, 1271, 1211, 1055, 1037, 852, 771, 719 cm^−1^. ESI-HRMS *m/z* [M+Na]^+^ calcd for C_29_H_54_NaN_4_O_2_ 513.4139, found 513.4125.

#### Methyl ((1-(3,7-Dimethylocta-2,6-Dien-1-yl)-1H-1,2,3-Triazol-4-yl)Methyl)-L-Prolinate (3m)

Compound **3m** was prepared from 30 mg (0.18 mmol) of N-propargyl methyl prolinate **1**, following the general procedure for the 1,3-dipolar cycloaddition, affording 47 mg of a yellow oil in 87 % yield. ^1^H NMR (300 MHz, CDCl_3_): δ 7.46 (s, 1H, C7-H), 5.41 (t, *J* = 7.2 Hz, 1H, C9-H), 5.08–5.01 (m, 1H, C13-H), 4.90 (t, *J* = 7.2 Hz, 2H, C8-H), 4.01 (d, *J* = 13.6 Hz, 1H, C6-H), 3.82 (d, *J* = 13.6 Hz, 1H, C6-H), 3.66 (s, 3H, OMe), 3.30 (dd, *J* = 8.1, 6.0 Hz, 1H, C2-H), 3.12–3.1 (m, 1H, C5-H), 2.54–2.46 (dt, *J* = 8.4, 8.2 Hz, 2H, C5-H), 1.7 (m, 6H, C3-H and C4-H), 1.65 (s, 3H, CH_3_), 1.63 (m, 6H, CH_3_), 1.55 (m, 3H, CH_3_). ^13^C NMR (75 MHz, CDCl_3_): δ 174.5 (COO), 144.4 (C6'), 122.7 (C7), 64.7 (C2), 53.4 (C5), 51.9 (OCH_3_), 50.4 (C8), 48.7 (C6), 31.9 CH_2_), 30.4 (CH_2_), 29.5 (C3), 29.3 (CH_2_), 29.1 (CH_2_), 26.5 (CH_2_), 23.1 (C4), 22.7 (CH_2_), 17.6 (CH_3_), 16.5 (CH_3_), 16.0 (CH_3_). IR (film): υ_max_ 3564, 3140, 2926, 2358, 1867, 1747, 1732, 1506, 1435, 1373, 1217, 1174, 1124, 1047, 844, 771 cm^−1^. ESI-HRMS *m/z* [M+H]^+^ calcd for C_19_H_35_N_4_O_2_ 351.2754, found 351.2746.

#### Methyl ((1-((2E,6E)-3,7,11-Trimethyldodeca-2,6,10-Trien-1-yl)-1H-1,2,3-Triazol-4-yl)Methyl)-L-Prolinate (3n) and Methyl ((1-((2E,6Z)-3,7,11-Trimethyldodeca-2,6,10-Trien-1-yl)-1H-1,2,3-Triazol-4-yl)Methyl)-L-Prolinate (3o)

Compound **3n** and **3o** were prepared from 50 mg (0.30 mmol) of N-propargyl methyl prolinate **1**, following the general procedure for the 1,3-dipolar cycloaddition, affording 53 mg of **3n** and 28 mg of **3o** as yellowish oils in 43 % and 23% yield, respectively.

**3n**-^1^H NMR (300 MHz, CDCl_3_): δ 7.47 (s, 1H, C7-H), 5.42 (t, *J* = 7.2 Hz, 1H, C9-H), 5.08–5.06 (m, 2H, C13-H and C16-H), 4.95 (t, *J* = 7.2 Hz, 2H, C8-H), 4.00 (d, *J* = 13.7 Hz, 1H, C6-H), 3.80 (d, *J* = 13.7 Hz, 1H, C6-H), 3.69 (s, 3H, OMe), 3.31 (dd, *J* = 8.7, 6.1 Hz, 1H, C2-H), 3.16–3.10 (m, 1H,C5-H), 2.61–2.48 (dt, *J* = 8.4, 8.2 Hz, 1H, C5-H), 1.70 (m, 4H, C3-H and C4-H), 2.12–1.91 (m, 8H), 1.78 (s, 3H, CH_3_), 1.65 (s, 3H, CH_3_), 1.63 (m, 3H, CH_3_), 1.55 (m, 3H, CH_3_). ^13^C NMR (75 MHz, CDCl_3_): δ 174.4 (COO), 144.4 (C6'), 143.1 (C), 135.7 (C), 131.3 (C), 124.2 (CH), 123.3 (CH), 122.0 (C7-H), 116.9 (CH), 64.7 (C2), 53.2 (C5), 51.8 (OCH_3_), 48.6 (C8), 47.8 (C6), 39.6 (CH_2_), 39.4 (CH_2_), 29.3 (C3), 26.6 (CH_2_), 26.1 (CH_2_), 25.6 (CH_2_), 22.9 (CH_3_), 17.6 (CH_3_), 16.5 (CH_3_), 16.0 (CH_3_). IR (film): υ_max_ 3417, 3124, 2992, 2358, 1867, 1747, 1732, 1539, 1456, 1317, 1271, 1122, 1047, 773 cm^−1^. ESI-HRMS *m/z* [M+H]^+^ calcd for C_24_H_39_N_4_O_2_ 415.3067, found 415.3067.

**3o**-^1^H NMR (300 MHz, CDCl_3_): δ 7.47 (s, 1H, C7-H), 5.42 (t, *J* = 7.2 Hz, 1H, C9-H), 5.08–5.06 (m, 2H, C13-H and C16-H), 4.95 (t, *J* = 7.2 Hz, 2H, C8-H), 4.00 (d, *J* = 13.7 Hz, 1H, C6-H), 3.80 (d, *J* = 13.7 Hz, 1H, C6-H), 3.69 (s, 3H, OMe), 3.31 (dd, *J* = 8.7, 6.1 Hz, 1H, C2-H), 3.16–3.10 (m, 1H, C5-H), 2.61–2.48 (dt, *J* = 8.4, 8.2 Hz, 1H, C5-H), 1.7 (m, 4H, C3-H and C4-H), 2.12–1.91 (m, 8H), 1.78 (s, 3H, CH_3_), 1.65 (s, 3H, CH_3_), 1.63 (m, 3H, CH_3_), 1.55 (m, 3H, CH_3_). ^13^C NMR (75 MHz, CDCl_3_): δ 174.4 (COO), 144.4 (C6'), 143.1 (C), 135.7 (C), 131.3 (C), 124.2 (CH), 123.3 (CH), 122.0 (C7-H), 116.9 (CH), 64.7 (C2), 53.2 (C5), 51.8 (OCH_3_), 48.6 (C8), 47.8 (C6), 39.6 (CH_2_), 39.4 (CH_2_), 29.3 (C3), 26.6 (CH_2_), 26.1 (CH_2_), 25.6 (CH_2_), 22.9 (CH_3_), 17.6 (CH_3_), 16.5 (CH_3_), 16.0 (CH_3_). IR (film): υ_max_ 3417, 3124, 2962, 2924, 2341, 1745, 1732, 1625, 1446, 1435, 1377, 1215, 1172, 1047, 775 cm^−1^. ESI-HRMS *m/z* [M+H]^+^ calcd for C_24_H_39_N_4_O_2_ 415.3067, found 415.3067.

#### Methyl ((1-((E)-3,7,11,15-Tetramethylhexadec-2-en-1-yl)-1H-1,2,3-Triazol-4-yl)Methyl) -L-Prolinate (3p) and Methyl ((1-(3,7,11,15-Tetramethylhexadec-2-en-1-yl)-1H-1,2,3-Triazol-4-yl)Methyl)-L-Prolinate (3q)

Compound **3p** and **3q** were prepared from 50 mg (0.30 mmol) of N-propargyl methyl prolinate **1**, following the general procedure for the 1,3-dipolar cycloaddition, affording 22 mg of **3p** (*E*-isomer) and 23 mg of **3q** (mixture *E:Z*) as yellowish oils in 38 % and 39 % yield, respectively.

**3p**-^1^H NMR (300 MHz, CDCl_3_): δ 7.47 (s, 1H, C7-H), 5.39 (t, *J* = 7.2 Hz, 1H, C9-H), 4.91 (t, *J* = 7.2 Hz, 2H, C8-H), 3.98 (d, *J* = 13.7 Hz, 1H, C6-H), 3.78 (d, *J* = 13.7 Hz, 1H, C6-H), 3.68 (s, 3H, OMe), 3.29 (dd, *J* = 8.7, 6.1 Hz, 1H, C2-H), 3.14–3.09 (m, 1H, C5-H), 2.54–2.46 (dt, *J* = 8.4, 8.2 Hz, 1H, C5-H), 2.11 (s, 3H, C10-CH_3_), 2.02–1.84 (m, 4H, C3-H and C4-H), 1.77–1.75 (m, 3H), 1.36–1.06 (m, 18H), 0.86–0.81 (m, 12H, CH_3_). ^13^C NMR (75 MHz, CDCl_3_): δ 174.4 (COO), 144.4 (C6'), 143.7 (C10), 122.1 (C7), 117.4 (C9), 64.8 (C2), 53.7 (C5), 51.9 (OCH_3_), 48.7 (C6), 39.8 (C8), 39.4 (CH_2_), 37.4 (CH_2_), 37.3 (CH_2_), 37.0 (CH_2_), 36.9 (CH_2_), 36.7 (CH_2_), 32.7 (CH_2_), 32.3 (CH_2_), 29.7 (C3), 29.4 (C4), 28.0 (CH_2_), 25.5 (CH_2_), 25.0 (CH_2_), 24.8 (CH_2_), 24.5 (CH_3_), 23.4 (CH_3_), 23.0 (CH_3_), 22.7 (CH_3_), 22.6 (CH_3_). IR (film): υ_max_ 3500, 3140, 2926, 2358, 1747, 1506, 1456, 1377, 1172, 1047, 933, 862, 775 cm^−1^. ESI-HRMS: mass calculated for C_29_H_52_N_4_NaO_2_ (M+Na)^+^, 511.3982, found 511.3970.

**3q**-^1^H NMR (300 MHz, CDCl_3_): δ 7.47 (s, 1H, C7-H), 5.39 (t, *J* = 7.2 Hz, 1H, CH), 4.91 (t, *J* = 7.2 Hz, 2H, C8-H), 3.98 (d, *J* = 13.7 Hz, 1H, C6-H), 3.78 (d, *J* = 13.7 Hz, 1H, C6-H), 3.68 (s, 3H, OMe), 3.29 (dd, *J* = 8.7, 6.1 Hz, 1H, C2-H), 3.14–3.09 (m, 1H, C5-H), 2.54–2.46 (dt, *J* = 8.4 Hz, *J* = 8.2 Hz, 1H, C5-H), 2.11 (s, 3H, CH_3_), 2.02–1.84 (m, 4H, C3-H and C4-H), 1.77–1.75 (m, 3H), 1.36–1.06 (m, 18H), 0.86–0.81 (m, 12H, CH_3_). ^13^C NMR (75 MHz, CDCl_3_): δ 174.4 (COO), 144.4 (C6'), 143.7 (C10), 122.1 (C7), 117.4 (C9), 64.8 (C2), 53.7 (C5), 51.9 (OCH_3_), 48.7 (C6), 39.8 (C8), 39.4 (CH_2_), 37.4 (CH_2_), 37.3 (CH_2_), 37.0 (CH_2_), 36.9 (CH_2_), 36.7 (CH_2_), 32.7 (CH_2_), 32.3 (CH_2_), 29.7 (C3), 29.4 (C4), 28.0 (CH_2_), 25.5 (CH_2_), 25.0 (CH_2_), 24.8 (CH_2_), 24.5 (CH_3_), 23.4 (CH_3_), 23.0 (CH_3_), 22.7 (CH_3_), 22.6 (CH_3_). IR (film): υ_max_ 3500, 3140, 2926, 2358, 1747, 1506, 1456, 1377, 1172, 1047, 933, 862, 775 cm^−1^. ESI-HRMS *m/z* [M+Na]^+^ calcd for C_29_H_52_N_4_NaO_2_ 511.3982, found 511.3970.

## Biology

### Reagents

All reagents were purchased from Sigma-Aldrich (St. Louis, MO, USA). Culture medium and fetal calf serum (FCS) were purchased from Cultilab (Campinas, SP, Brazil).

### Cells and Parasites

*T. cruzi* CL strain clone 14 epimastigotes (Brener and Chiari, 1965) were maintained in the exponential growth phase by subculturing every 48 h in Liver Infusion Tryptose (LIT) medium supplemented with 10% FCS (Vitrocell, Campinas, São Paulo, Brazil) at 28 °C.

### Growth Inhibition Assays

*T. cruzi* epimastigotes in the exponential growth phase (5.0-6.0 x 10^7^ cells mL^−1^) were cultured in fresh LIT medium. The cells were treated with different concentrations of drugs or not treated (negative control). A combination of Rotenone (60 μM) and Antimycin (0.5 μM) was used as a positive control for inhibition as previously described. (Magdaleno et al., 2009) The cells (2.5 x 10^6^ mL^−1^) were transferred to 96-well culture plates and incubated at 28 °C. Cell proliferation was quantified by reading the optical density (OD) at 620 nm for 8 days. The OD was converted to cell density values (cells per mL) using a linear regression equation previously obtained under the same conditions. The concentration of compounds that inhibited 50% of parasite proliferation (IC_50_) was determined during the exponential growth phase (4 days) by fitting the data to a typical sigmoidal dose-response curve using OriginPro8. The compounds were evaluated in quadruplicate in each experiment. The results shown here correspond to three independent experiments. As a cell growth inhibition control, growth curves in which 200 mM rotenone and 0.5 mM antimycin were added to the culture medium were run in parallel for all experiments.

### Cytotoxicity Assay

To evaluate the analogs toxicity, Vero cells previously plated on 96 multi-well plate in DMEM 2% FBS and incubated for 48 h at 37 °C in a humid atmosphere containing 5% CO_2_, were incubated with 700 μL of DMEM 2% FBS supplemented with each analog for 48 h. The concentration (μM) was different with each analog:

◦ **3i**: 20 μM, 40 μM, 60 μM, 80 μM, 100 μM and 120 μM.
◦ **3k**: 5 μM, 10 μM, 15 μM, 20 μM, 25 μM and 30 μM.
◦ **3n**: 5 μM, 10 μM, 15 μM, 20 μM, 25 μM and 30 μM.
◦ DMSO: 5 μM, 25 μM, 50 μM and 100 μM.
◦ Benznidazole: 10 μM, 100 μM, 200 μM and 300 μM.

The viability of cells was measured by MTT dye (3-(4,5-Dimethylthiazol-2-yl)-2,5-diphenyltratazolium bromide, Sigma-Aldrich) colorimetric method. Benznidazole and DMSO were used as positive and negative controls, respectively. Data are expressed as means ± SD of the results of three independent assays of each condition.

### L-Proline Transport Inhibition Assay

Parasites in exponential growth phase were washed three times by centrifugation and resuspended in phosphate buffered saline (PBS), pH 7.4. Cells were counted in a Neubauer chamber, adjusted with the same buffer to a final density of 200 × 10^6^ cells/mL and distributed in aliquots of 100 μL (containing 20 × 10^6^ cells each). Transport assays were initiated by the addition to the assay tubes of 100 μL of the desired dilution of L-proline in PBS in the presence of 0.5 mCi of L-[3H] proline. Unless otherwise specified, V0 was measured at 28 °C for 30 s by incorporation of 0.75 and 3 mM L-proline traced with 1 μCi of U-^14^C-L-Pro (Perkin Elmer). In all cases, proline transport was stopped by addition of 800 mL of cold 50 mM proline in PBS (pH 7.4), and rapid washing by centrifugation at 10,000x *g* for 2 min. Background values in each experiment were measured by simultaneous addition of radiolabeled L-proline and stop solution as previously described (Silber et al., 2002).

### Competition Assay

For the competition assays, the transport experiments were performed as described above using the L-Pro concentration corresponding to the *K*_*m*_ (0.31 mM). Those conditions were chosen considering an inhibitory activity by structurally related compounds should be competitive, and so, should be evidenced by a change in the *V*_*max*_ at L-Pro concentrations close to the *K*_*m*_. Non-competitive inhibitors, if any, should diminish *V*_*max*_, which is measured at 10 x *K*_*m*_ L-Pro (3.1 mM) (Silber et al., 2002).

### Statistical Analysis

One-way ANOVA followed by the Tukey test was used for statistical analysis. The *T*-test was used to analyze differences between groups. P < 0.05 was considered statistically significant.

## Results

### Synthesis of L-Proline Transport Inhibitors

**Figure 1:**
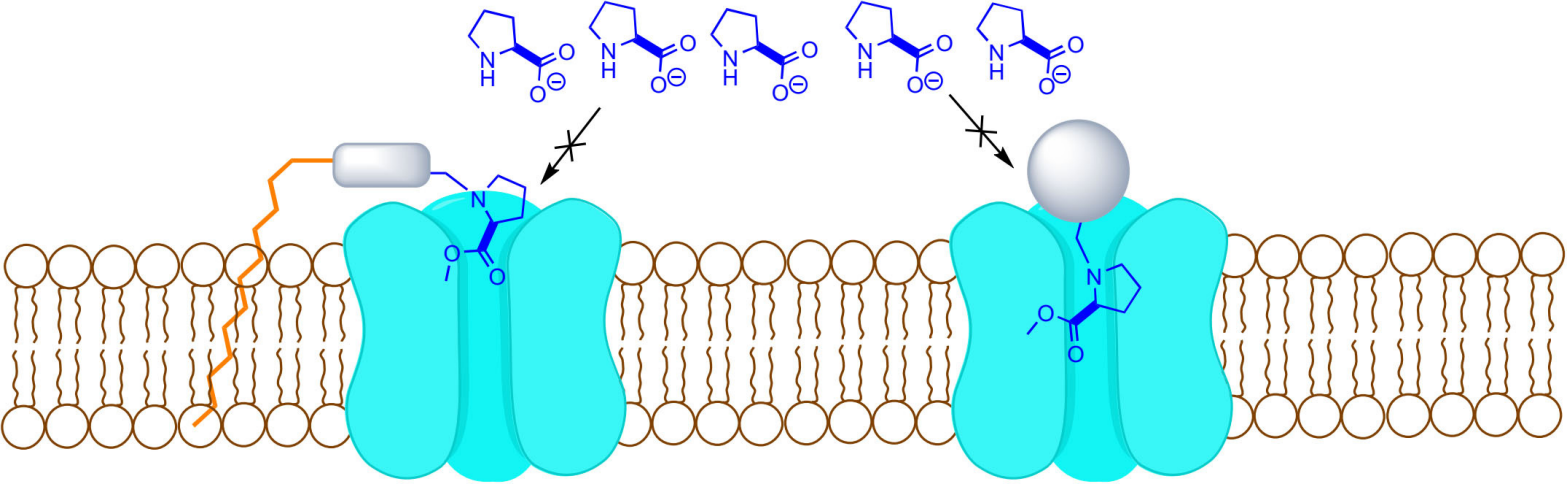
Proposed model for transport inhibition.

A proper uptake blocker needs to be recognized by the transporter, but not being able to go through, by adding a bulky substituent or a membrane interacting portion that prevents its transport (Figure 1).

**Figure 2:**
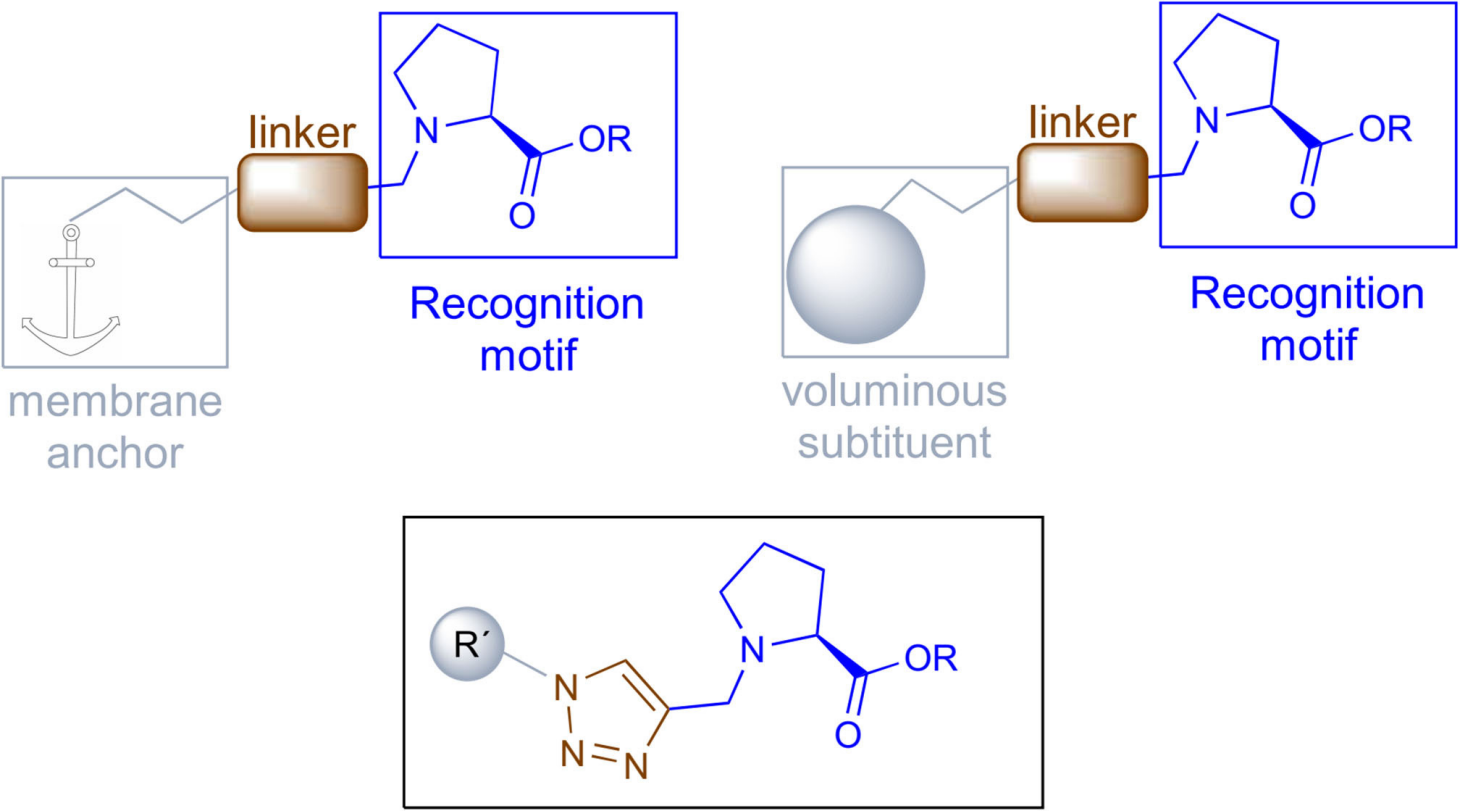
Design of proline transport inhibitors.

This model will require a compound holding a recognition motif, a linker and a membrane anchor or a voluminous substituent to block the L-proline incorporation. The recognition portion will have L-proline to specifically interact with the transporter, the membrane anchor should be a non-polar group and both parts will be connected by a 1,2,3-triazole (Figure 2).

**Scheme 1:**
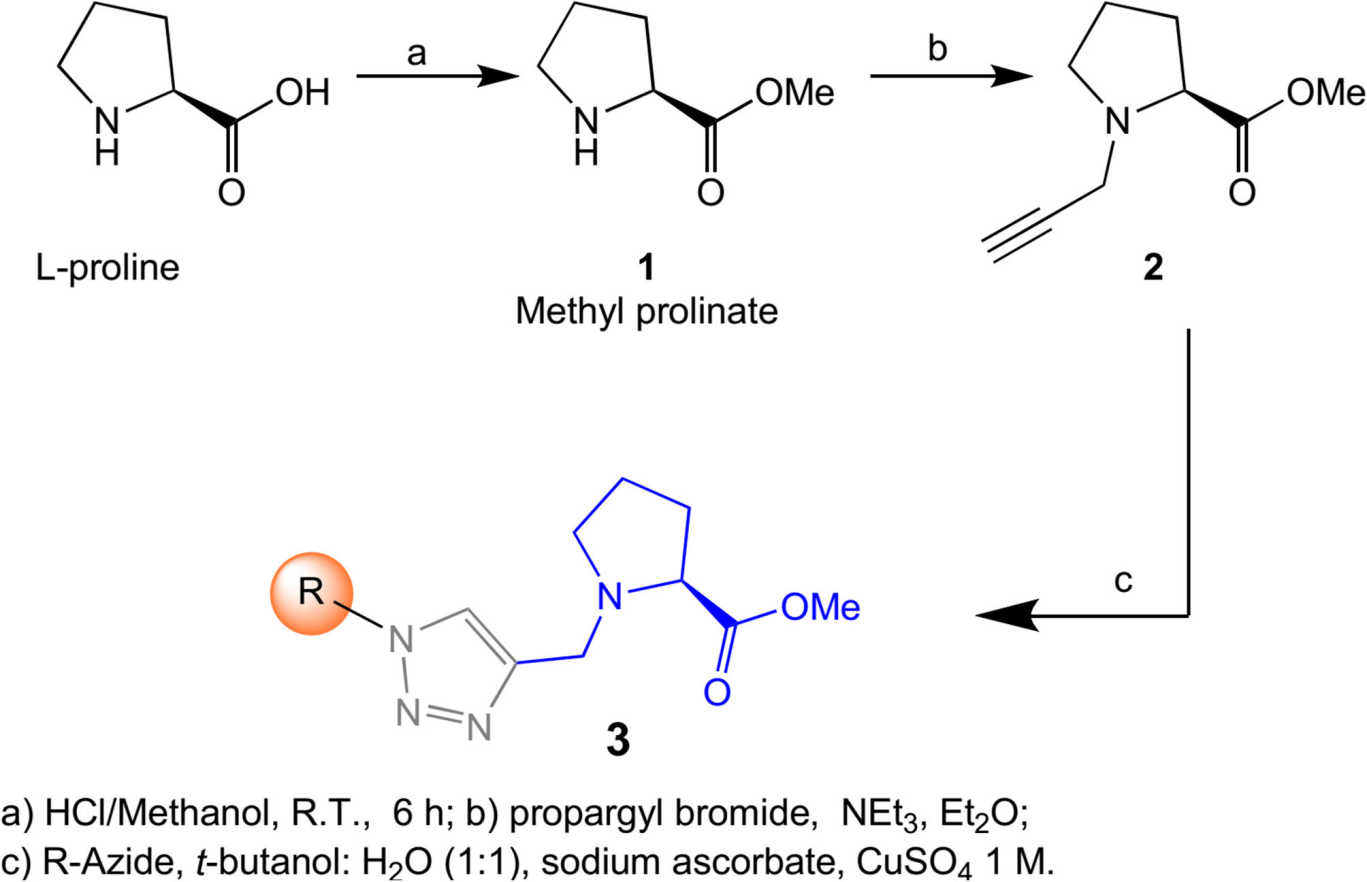
Synthesis of proline transport inhibitors.

To validate the proposed model, we proceeded to make a small library following the synthetic strategy shown on Scheme 1. The synthesis started from commercial L-proline, preparing the key intermediate in two steps, including an esterification to produce the methyl prolinate **1** followed by an N-alkylation with propargyl bromide. Once the required key propargyl methyl prolinate **2** intermediate was prepared, a pool of different azides covering a variety of steric moieties including, aryl, alkyl and isoprenyl substituents was selected to explore their capacity to interact with the membrane. Azides were prepared by direct substitution of bromide with sodium azide on DMF except for geranyl-, farnesyl-, and phytylazides, which were synthesized from geraniol, farnesol and phytol, respectively, using diphenylphosphorylazide (DPPA) following Thompson's procedure (Thompson et al., 1993). Phytylazide was found to be a mixture of three chemical entities in equilibrium: tertiary azide, *E* and *Z* isomers of the primary azide, following the same behavior previously observed on geranyl-, and farnesylazide (Porta et al., 2014). Allylic azides can be obtained as a mixture, because they exist as equilibrating mixtures of regioisomers due to the [3,3] sigmatropic rearrangement (Winstein rearrangement) (Gagneuz et al., 1969). That was the case of geranyl, farnesyl and phytylazide, a mixture of primary:tertiary (8:1), being the primary as 1.6:1 (*E*:*Z*) ratio (Porta et al., 2017b).

**Figure 3:**
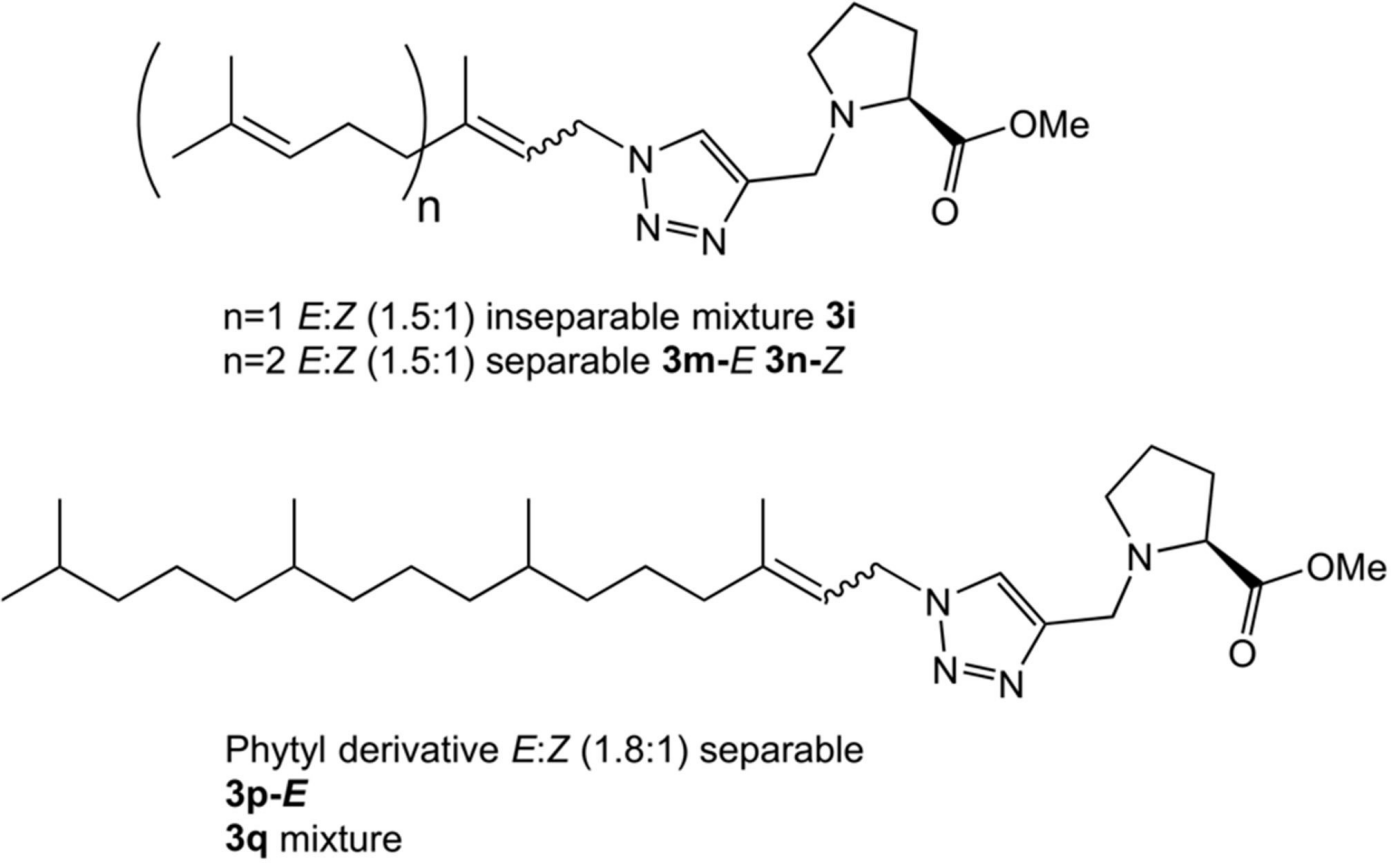
Prenyl analogs prepared.

Having prepared the pool of azides, we continued with the synthesis of a focused library of inhibitors through click chemistry. Reactions were conducted in a parallel solution synthesis setup under copper (II) sulfate catalytic conditions in water:*t*-BuOH (1:1), using sodium ascorbate as a reductant (Rostovtsev et al., 2002; Labadie et al., 2011; Porta et al., 2017a). In general, reactions needed an excess of azides for completion and a reaction time was 18 h. All the products have 1,4-substitution on the 1,2,3,-triazol as expected, based on the original description of this methodology and our previous work (Porta et al., 2014, 2017a). Reactions with aliphatic and benzylic azides produced a single product, with yields that are slightly better for the last ones. The reaction with the mixture of geranyl azides generated **3i**, which was identified as an inseparable mixture of *E* and *Z* isomers (^1^H NMR, 1.5:1), in accordance with our previous results (Porta et al., 2017a). When farnesyl azide was used, a mixture of regioisomers was also obtained with the same ratio, but in this case they were separable (Figure 3). When phytylazide was used the same regioisomers were isolated after the reaction with N-propargyl methyl prolinate **1**, presenting a higher *E*:*Z* ratio (1.8:1). Extensive purification work allowed only the isolation of the *E*-isomer of a pure compound (**3p-*E***, Figure 3), leaving the remaining product **3q** as a mixture *E*:*Z*.

Final products **3a-3q**, presented in Table 1, were obtained with an average 67 % yield after purification and were completely characterized by 1D and 2D NMR and ESI-HRMS.

**Table 1:** Anti-*T. cruzi* activity of the proline derivatives.

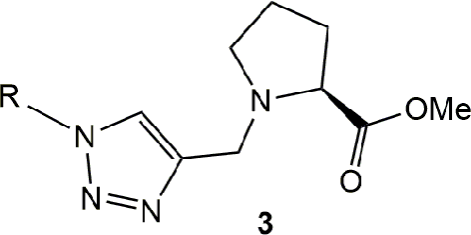			
**Compound**	**R**	**Yield (%)**	***T. cruzi*^a^ IC_50_ [μM]**
**3a**	CH_2_COOEt	80	>100
**3b**	(CH_2_)_4_COOEt	69	>100
**3c**	Bn	80	>100
**3d**	Cyclohexyl	39	>100
**3e**	Ph-CH_2_CH_2_CH_2_	82	>100
**3f**	Cinnamyl	60	>100
**3g**	CH_2_-naphtyl	81	100
**3h**	Octyl	81	>100
**3i**	Decyl	75	38.97 ± 1.37
**3j**	Cetyl	42	24.07 ± 0.66
**3k**	Oleyl	59	35.06 ± 6.96
**3l**	Eicosanyl	58	100
**3m**	Geranyl	87	>100
**3n**	*E*-Farnesyl	43	48.32 ± 1.29
**3o**	*Z*-Farnesyl	23	58.60 ± 1.37
**3p**	*E*-Phytyl	38	69.75 ± 2.17
**3q**	Phytyl-Mixture	39	48.27 ± 5.81
**Benznidazole**			7.00 [57]

a *Epimastigotes CL14, results shown are means (SD) from the three independent experiments*.

In order to evaluate the biological activity of the prepared collection we decided to initially determine the activity on *T. cruzi* epimastigotes. Then, to validate the L-proline transporter as the molecular target, the intracellular concentration of the amino acid was measured in competition assays with compounds that shown the best antiparasitic activity. Finally, the cytotoxicity of the selected candidates was evaluated in African green monkey kidney epithelial (VERO) cells to estimate the selectivity toward the parasite.

### *In vitro* Activity Against *T. cruzi* Epimastigotes

The compounds collection was assayed against *T. cruzi* epimastigotes (CL strain clone 14) (Brener and Chiari, 1965) at a maximum concentration of 100 μM. Eight compounds of the total list did not affect the parasite growth at that concentration (**3a**, **3b**, **3c**, **3d**, **3e**, **3f**, **3h**, **3m**), being considered inactive. (Table 1) The naphtyl derivative **3g** and the eicosanyl analog **3l** have an IC_50_ of 100 μM. A second group of active members of the collection includes the remaining prenyl derivatives **3n**, **3o**, **3p** and **3p** with IC_50_s 48.32, 58.60, 69.75, 48.27 μM, respectively. (Table 1) The remaining group contains the aliphatic derivatives **3i**, **3j** and **3k**. Those compounds have IC_50_s below 40 μM, being the most active members the collection. The decyl and the oleyl derivatives **3i** and **3k** have similar activity (IC_50_ of 38.97 and 35.06 μM, respectively), while the cetyl analog **3j** has a IC_50_ of 24.07 μM, considerably slower than the other two (Table 1).

### *In vitro* Cytotoxicity Assay on Vero Cells

Vero cells are well established model to test cytotoxicity *in vitro* because it is an aneuploid and a continuous cell linage. Initially the library was screened at a fix concentration of 4.75 μg/mL and none of the analogs showed cytotoxic activity. The most active analogs of the series, compounds **3i**, **3j**, **3k**, and **3n**, were submitted to a further analysis to determine their IC_50_. Compound **3j** was not soluble at 50 μM which made not possible to calculate its IC_50_. Analogs **3i**, **3k**, and **3n** were soluble in a concentration range allowing to perform the assay, showing IC_50_ of 43 μM, 17 μM and 14 μM, respectively.

### Proline Transport Assay

**Figure 4:**
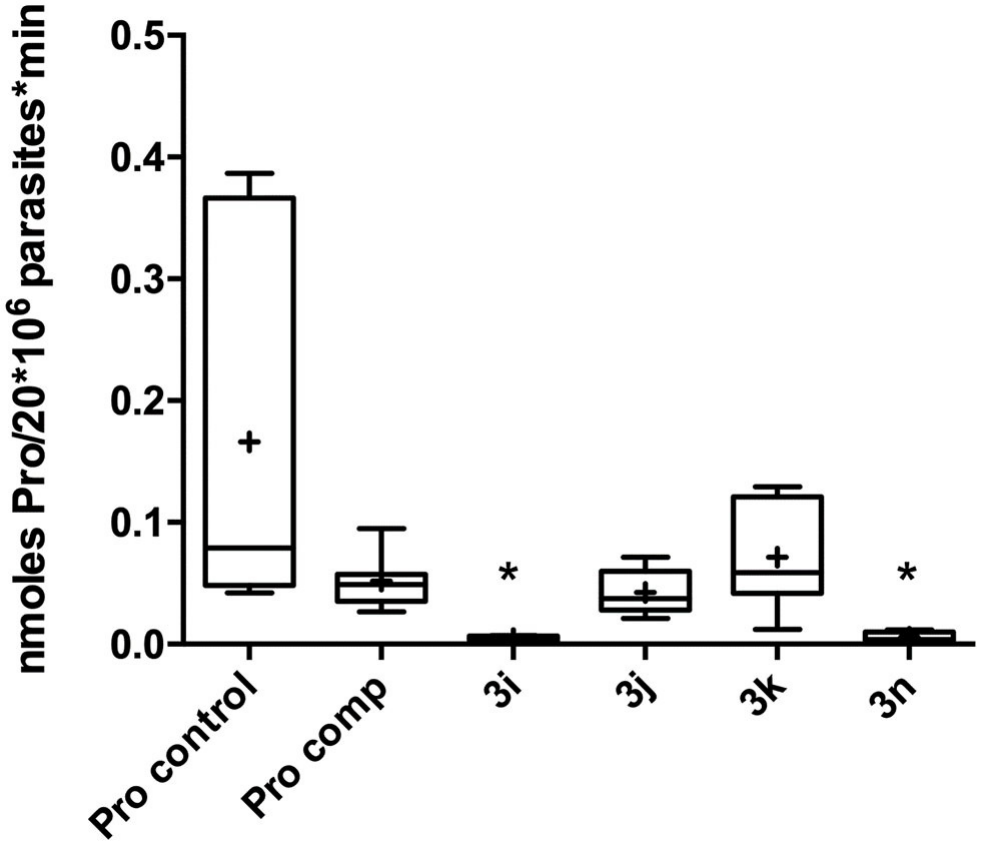
Proline uptake assay. Pro control = Proline control, Pro comp = L-Proline as competitor. **3i-k** and **3n** = proline level incubated (*p < 0.05) with selected analogs. Pro control (0.31 mM L-proline, [^3^H] proline, PBS). Pro Comp (3.1 mM L-proline, [^3^H] proline, PBS). Compounds **3i-k** and **3n** (0.31 mM L-proline, 3.1 mM analog **3x**, [^3^H] proline, PBS). Stop solution: 50 mM L-proline added after 30 s.

In order to obtain a deeper insight into the molecular mechanism of the most active compounds, we performed a proline transport competition assay. The compounds with an IC_50_ lower than 50 μM (**3i**, **3j**, **3k**, and **3n**) were selected to perform the proline uptake assay aiming to determine the transport inhibition (Magdaleno et al., 2009). The analogs were assayed on *T. cruzi* epimastigotes incubated with proline at the transporter Km concentration (0.31 mM), and the analogs were assayed at concentration 10-fold higher (3.1 mM). Surprisingly, we observed that the compounds with higher activity on *T. cruzi* epimastigotes (lower IC_50_ values) showed inhibition but they were not strong enough when compared to analogs with lower antiparasitic activity (higher IC_50_ values**)**. As can be seen on Figure 4, compounds **3i** (*T. cruzi* epimastigotes IC_50_= 38 μM) and **3n** (*T. cruzi* epimastigotes IC_50_= 49 μM) showed a proline transport inhibition higher than 75% being more active than the analog **3j** that only produced an inhibition of 20%. The oleyl derivate (**3k**), with an unsaturated fatty tail, has a similar IC_50_ on *T. cruzi* epimastigotes compared to the cetyl analog (**3j**) showing no inhibition in terms of proline uptake (Table 2).

**Table 2:** Proline uptake inhibition of selected analogs.

**Compound**	**R**	***T. cruzi* IC_50_ [μM]**	**% of Proline inh**.
**3i**	Decyl	38.97 ± 1.37	87
**3j**	Cetyl	24.07 ± 0.66	18^#^
**3k**	Oleyl	35.06 ± 6.96	0
**3n**	*E*-Farnesyl	48.32 ± 1.29	99

# *Precipitation was observed over the experiment*.

## Discussion

The selective inhibition of transporters has been proposed as a valuable target to develop new medications against different pathologies including neurological disorders (Qosa et al., 2016) and parasitic diseases (Sayé et al., 2019). The specific inhibition of neuronal glycine, alanine, serine, and cysteine transporters have been studied as molecular targets for new treatment of schizophrenia (Pinard et al., 2010; Schneider et al., 2012; Carland et al., 2014). A high-affinity transporter for proline has been identified, providing an important evidence for proline as a neurotransmitter (Hauptmann et al., 1983). A high throughput screening campaign for high affinity proline transporter inhibitors resulted in the identification of the selective inhibitor LP-403812 (Yu et al., 2009). Summarizing, the participation of metabolites uptake in critical processes in health and diseases has been well demonstrated.

**Figure 5:**
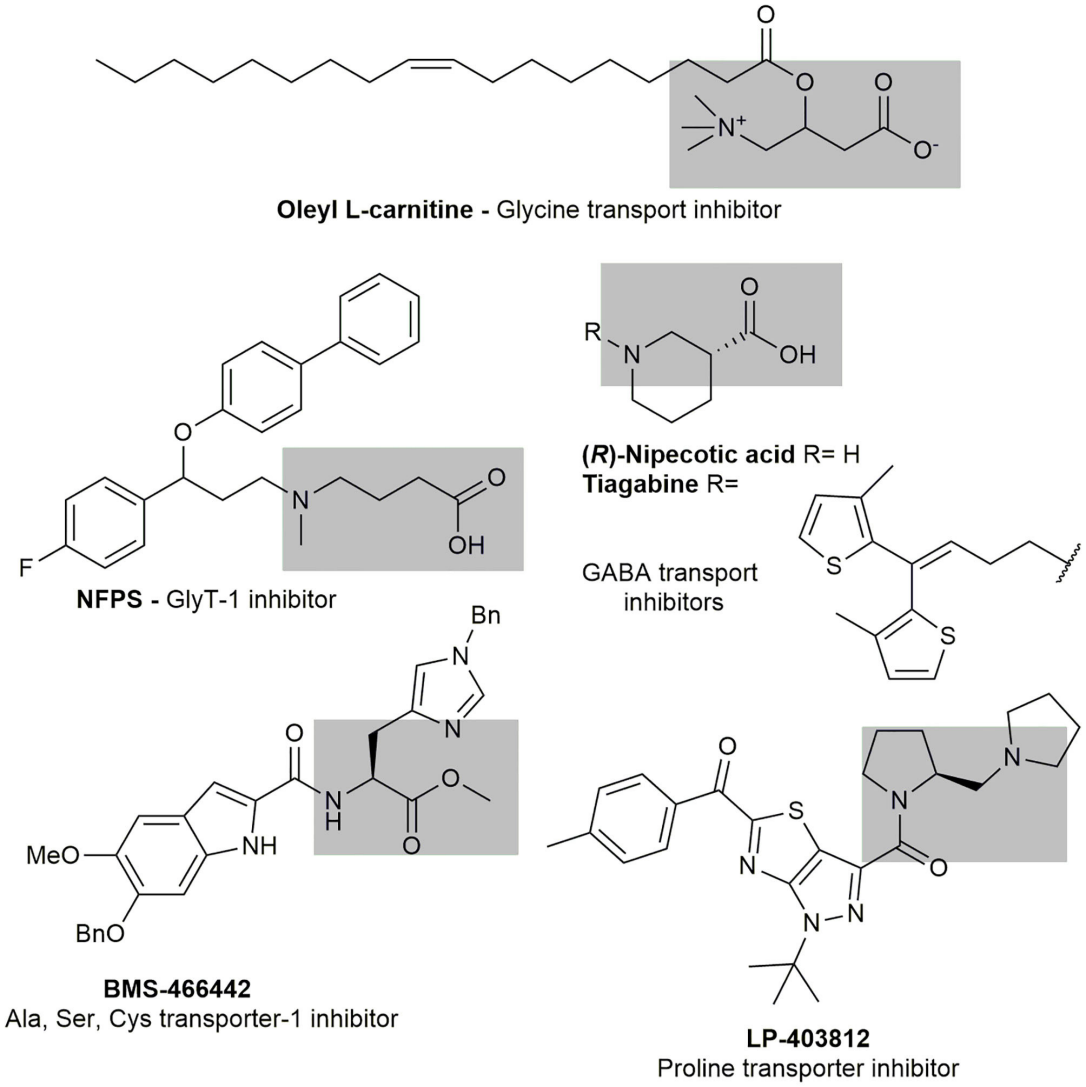
Known amino acid transport inhibitors.

A systematic design pipeline for molecules targeting molecular transporters has not been properly explored. The discovery of most of transporters inhibitors happened by chance or through large HTS campaigns. Burns et al. (2009) pioneered a work in this sense proposing a polyamine-fatty acid conjugate as a polyamine transporter inhibitor. In their rational, the polyamine portion is recognized by the transporter and the fatty acid interacts with the membrane, blocking the polyamine entrance. The newly designed inhibitors (L-Lyz(C18-Acyl-spermine) combined with DMFO display selective antitumoral activity. All the inhibitors mentioned before contained an amino acid portion, or an amino acid mimic, that is recognized by the transporter as a common feature (Figure 5).

With those precedents in mind, we proposed/presented here a general model for aminoacid transporter inhibitors. Our model included an aminoacid portion for recognition, linked to a variable region with different lipophilic and bulk substituents.

We identified the 1,2,3-triazole as a proper linker for our transport inhibitors. The neutral nature of this heterocycle has properly suited the requirements for bioconjugation, protein labeling and immobilization (Gauchet et al., 2006; Mckay and Finn, 2014) and for combining different pharmacophores to make hybrid compounds or chimeras. The Cu(I) azide-alkyne reaction, the quintessence of click chemistry, had a strong impact in many research areas including Medicinal Chemistry (Tron et al., 2008; Agalave et al., 2011). The easy reaction conditions has made this methodology very useful to rapidly prepare libraries of compounds, including antiparasitic drug candidates (Carvalho et al., 2010; Hamann et al., 2014; Porta et al., 2017a).

Using 1,2,3-triazole as linker, we prepared products introducing substituents with proper membrane anchor properties. Fatty acids and isoprenyl chains are selectively introduced in proteins by post translational modifications and served as a mediator for membrane association increasing their molecular hydrophobicity (Hannoush and Sun, 2010). Looking to produce similar anchoring properties, isoprenyl and linear long alkyl chain were selected as some of the 1,2,3-triazole substituents, introduced as azide on the heterocycle. Additionally, azides with bulky substituent were also used looking to obstruct the transporter. A library of 17 compounds was finally prepared including two alkyl ester, five alicyclic and aryl, five alkyl and five prenyl derivatives.

The proline uptake systems have similar biochemical characteristics in epimastigotes and the mammalian *T. cruzi* stages (Silber et al., 2002; Tonelli et al., 2004). It has been demonstrated that these stages are sensitive to proline availability (Tonelli et al., 2004) and uptake (Magdaleno et al., 2009). With those precedents in mind, we decided to evaluate the activity on epimastigotes. The results of the inhibition on *T. cruzi* epimastigotes shown that half of the compounds prepared did not show activity at the maximum concentration tested of 100 μM. Between the inactive analogs, **3a** and **3b** holds the shortest substituents of the library and have esters on the side-chain. Being the most polar substituent of the series and susceptible to hydrolysis, seems to be a possible explanation for the lack of activity. Analogs **3c**, **3d**, **3e**, and **3f**, contain non-polar cyclic substituents either arylic or alicyclic, were also inactive. Interestingly, the naphtyl derivative **3g**, the bulkier member of the collection, has an IC_50_ of 100 μM. Together those results shown that polycyclic bulky substituents could be required to improve the activity. Nevertheless, the fact that the aromatic analogs were inactive discouraged the idea that π-stacking interactions are involved on the binding to the molecular target.

As was mentioned before, fatty acids and isoprenyl chains contribute as mediators for protein membrane anchoring, therefore, ten analogs were included with those substituents. The hypothesis that this kind of substituents contribute to improve the activity, seems to be validated because only the analogs holding the shorter substituents, **3h** (R=octyl) and **3m** (R=geranyl) were inactive. A detailed look at the aliphatic derivatives' activities did not show a direct correlation between the IC_50_ and the chain length. The activity increases from octyl **3i** to decyl **3h** derivative (IC_50_ > 100 and 38.27 mM, respectively), being the cetyl analog **3j**, the most active (IC_50_ 24.54 mM). Then, the activity decreases to 35.06 mM for the oleyl derivative **3k** and 100 mM for the eicosanyl analog **3l**. A similar behavior is observed for the prenyl derivatives, but in this case the difference is less pronounced. Moving from the geranyl derivative **3h** to the farnesyl analogs the activity increase to 48.32 mM for the *E*-isomer **3n** that is slightly more active than the *Z*-isomer **3o** (59.60 mM). Then, as happened with the alkylated analogs, the activity decreases for the longer member of the family, the phytyl derivatives **3p** (*E*-isomer) and **3q** (E/Z mixture), with IC_50_s 69.75 and 48.27 mM, respectively.

**Figure 6:**
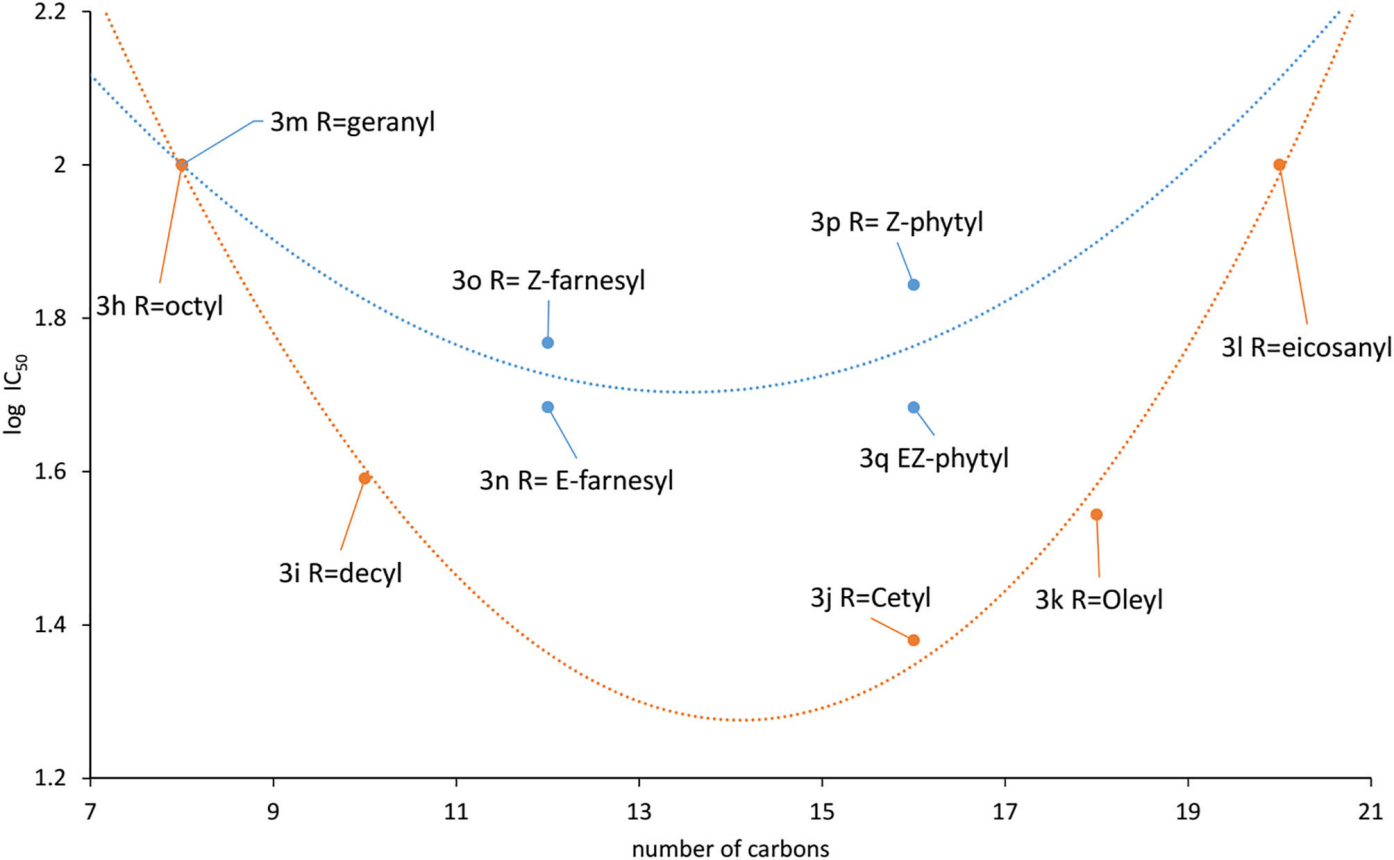
Correlation between activity and substituents chain length (blue: prenyl, orange: aliphatic).

A tendency can be visualized when the log IC_50_ was plotted against the carbon chain length and the aliphatic and the prenylated are separately correlated (Figure 6). One interesting outcome of this chart is that both curves have their minimum around 14 carbon atoms, that appears to be the optimal chain length for the activity.

These comparisons of the compounds have shown that most of them are considerable more active than L-thiazolidine-4-carboxylic acid (T4C), the only reported antichagasic proline derivative, with an IC_50_ of 890 μM on *T. cruzi* epimastigotes (Magdaleno et al., 2009). Analogs **3j**, **3i**, **3k**, **3n** were 36, 23, 22, and 18 times more active than T4C. That marked difference on the activity highlight the importance of the proline ring decoration on the antiparasitic activity.

The cytotoxicity of most active compounds (analogs **3i**, **3k**, and **3n**) were tested in cultured monkey kidney Vero cells to estimate the selectivity toward the parasite. The IC_50_ were 43 μM, 17 μM and 14 μM, respectively, being an adequate concentration window for future studies in intracellular stages. The selectivity index, (calculated as IC_50_Vero cells / IC_50_ *T. cruzi* epimastigotes) were 1.13, 0.43 and 0.25 for compounds **3i**, **3k**, and **3n**, respectively. This numbers shown a similar susceptibility to the mentioned compounds. The toxicity displayed may be linked to the proline transport inhibition, but that hypothesis must be validated. These results are not promising at this point to propose that compounds of our collection could be good candidates for anti-chagasic drug development. Nevertheless, the applied strategy settles the basis to design inhibitors against *T. cruzi* biological targets in both, the insect, and the mammalian stages.

Finally, to validate the L-proline transporter as the molecular target, the intracellular concentration of the amino acid was measured in competition assays with compounds that shown the best antiparasitic activity. Analogs **3i**, **3k**, and **3n** were assayed. Interestingly, the proline uptake inhibition of those compounds did not follow the antiparasitic activity. The first hypothesis to explain that behavior was based on the presence of unsaturations on fatty tail. The difference between **3i** and **3k** relay on the unsaturation on the oleyl chain of the last. The structure of **3k** shows a twisted conformation produced by the C9–double bond that also restricts the rotation around the neighbor bonds. Furthermore, the tail length of **3i** and **3k** differ in 6 carbon and the most active is the decyl analog (**3i**) in terms of transport inhibition, matching the behavior displayed in Figure 6. The *E*-farnesyl derivative **3n** has a trimethyl substituted side-chain that is twelve carbon long, with three double bonds. Interestingly, this analog produced a complete inhibition of the proline uptake but is the less active of this series against *T. cruzi*. (Table 2) The fact that this analog was a superior inhibitor of the transporter could be the result of a better binding. Also, it could be attributed to the markedly different conformation of the analog due to the restricted rotation of the isoprenyl chain. Those restrictions should contribute to block the transporter once the proline region is recognized (Sayé et al., 2017). The inhibition of proline uptake by analogs **3i** and **3n**, resulted considerably more active than that inhibition by T4C (Magdaleno et al., 2009). Those differences were clearly related with the N1-alylated-1,2,3-triazolyl chain introduced on the proline.

## Conclusions

In the present study, a strategy to design amino acids transport inhibitors was proposed. The uptake blocker is composed by a recognition motif, a linker and a bulky substituent or a membrane interacting portion. A set of seventeen 1,5-subtituted-1,2,3-triazole derivatives of methyl prolinate were prepared to validate the design. They were initially assayed against *T. cruzi* epimastigotes showing comparable potency than the control drug benznidazole. The antiparasitic activity profile of the series allowed us to establish a well-defined structural-activity relationship were the nature of the side-chain play a critical role. In order to validate the design, the inhibition of the proline uptake was studied with the analogs **3i**, **3j**, **3k**, and **3n** that displayed the best antiparasitic activity. The analogs with **3i** (R=decyl) and **3n** (R=*E*-farnesyl) produced a markedly reduction of the internalized proline. Those studies are strong evidence to validate our design of the transporter inhibitor that also linked the antiparasitic activity with the proline uptake.

The proline uptake has been explored as target for Chagas disease by drug repurposing. Using that approach, crystal violet has been identified as an interesting candidate (Sayé et al., 2020). Our approach is the first report of new compounds that have been design, prepared and validated as proline uptake blockers with antiparasitic activity. Unfortunately, the most active products displayed low selectivity toward the parasite, not being good candidates for future development as antichagasic drugs. Nevertheless, a complete study on the other *T. cruzi* life cycle stages could confirm or discard that hypothesis. The validated design of aminoacid transport inhibitor should open new applications on the study of physiological role of amino acid transporters in the central nervous system.

## Data Availability Statement

The raw data supporting the conclusions of this article will be made available by the authors, without undue reservation.

## Author Contributions

LF and EP-Z were responsible for the synthesis, purification and structural characterization of all the products. LF and MB performed the antiproliferative assays against *Trypanosoma cruzi*, carried out the proline transport inhibition assays, and interpreted the results. LF and LP performed the cytotoxicity assays on Vero cells. GL wrote most of the manuscript and supervised the study. GL, AS, and JC were the project leaders organizing and guiding experiments. All authors contributed to refining the manuscript and approved the final manuscript.

## Conflict of Interest

The authors declare that the research was conducted in the absence of any commercial or financial relationships that could be construed as a potential conflict of interest.
