# Supplementary material for: Targeting L-Proline Uptake as New Strategy for Anti-chagas Drug Development
Source: Front Chem. 2020 Aug 25;8:696. doi: 10.3389/fchem.2020.00696 (PMC7477874; doi:10.3389/fchem.2020.00696)
Supplement: Supplementary file 1 [file Table_1.docx]

Targeting L-proline uptake as new strategy for anti-chagas drug development

Lucía Fargnoli^1^, Esteban A. Panozzo-Zénere^1^, María Julia Barisón^2^, Lucas Pagura^3^, Julia A. Cricco^3^, Ariel M. Silber^2^ and Guillermo R. Labadie^1,*^

^1^Instituto de Química de Rosario (IQUIR), Facultad de Ciencias Bioquímicas y Farmacéuticas-Universidad Nacional de Rosario-Rosario, Argentina.

^2^Laboratory of Biochemistry of Tryps-LaBTryps-Departamento de Parasitologia, Instituto de Ciências Biomédicas, Universidade de São Paulo, Cidade Universitária, São Paulo, Brasil.

^3^Instituto de Biología Molecular y Celular de Rosario (IBR), Consejo Nacional de Investigaciones Científicas y Técnicas CONICET–Facultad de Ciencias Bioquímicas y Farmacéuticas, Universidad Nacional de Rosario, Rosario, Argentina.

Corresponding author: labadie@iquir-conicet.gov.ar

# Spectral data of **compound 3a**

**^1^H NMR (300 MHz, CDCl_3_)**

**^13^C NMR (75 MHz, CDCl_3_)**

Spectral data of **compound 3b**

**^1^H NMR (300 MHz, CDCl_3_)**

**^13^C NMR (75 MHz, CDCl_3_)**

# Spectral data of **compound 3c**

**^1^H NMR (300 MHz, CDCl_3_)**

**^13^C NMR (75 MHz, CDCl_3_)**

Spectral data of **compound 3d**

**^1^H NMR (300 MHz, CDCl_3_)**

**^13^C NMR (75 MHz, CDCl_3_)**

**Spectral data of compound 3e**

**^1^H NMR (300 MHz, CDCl_3_)**

**^13^C NMR (75 MHz, CDCl_3_)**

# Spectral data of **compound 3f**

**^1^H NMR (300 MHz, CDCl_3_)**

**^13^C NMR (75 MHz, CDCl_3_)**

# Spectral data of **compound 3g**

**^1^H NMR (300 MHz, CDCl_3_)**

**^13^C NMR (75 MHz, CDCl_3_)**

# Spectral data of **compound 3h**

**^1^H NMR (300 MHz, CDCl_3_)**

**^13^C NMR (75 MHz, CDCl_3_)**

# Spectral data of **compound 3i**

**^1^H NMR (300 MHz, CDCl_3_)**

**^13^C NMR (75 MHz, CDCl_3_)**

Spectral data of **compound 3j**

**^1^H NMR (300 MHz, CDCl_3_)**

**^13^C NMR (75 MHz, CDCl_3_)**

# Spectral data of **compound 3k**

**^1^H NMR (300 MHz, CDCl_3_)**

**^13^C NMR (75 MHz, CDCl_3_)**

# Spectral data of **compound 3l**

**^1^H NMR (300 MHz, CDCl_3_)**

**^13^C NMR (75 MHz, CDCl_3_)**

# Spectral data of **compound 3m**

**^1^H NMR (300 MHz, CDCl_3_)**

**^13^C NMR (75 MHz, CDCl_3_)**

# Spectral data of **compound 3n**

**^1^H NMR (300 MHz, CDCl_3_)**

**^13^C NMR(75MHz, CDCl_3_)**

# Spectral data of **compound 3o**

**^1^H NMR (300 MHz, CDCl_3_)**

**^13^C NMR (75 MHz, CDCl_3_)**

# Spectral data of **compound 3q**

**^1^H NMR (300 MHz, CDCl_3_)**

**^13^C NMR (75 MHz, CDCl_3_)**
